# A Study on the Behavior of Smart Starch-*co*-poly(*N*-isopropylacrylamide) Hybrid Microgels for Encapsulation of Methylene Blue

**DOI:** 10.1021/acsomega.4c01947

**Published:** 2024-06-11

**Authors:** Andresa da Costa Ribeiro, Tania T. Tominaga, Taiana G. Moretti Bonadio, Nádya P. da Silveira, Daiani C. Leite

**Affiliations:** † Applied Physics in Materials Group, Departamento de Física, Universidade Estadual do Centro-Oeste, Guarapuava, PR 85040-167, Brazil; ‡ Post Graduation Program in Chemistry (PPGQ), Chemistry Institute, Universidade Federal do Rio Grande do Sul, Porto Alegre, RS 91501-970, Brazil; § Laboratório de Superfícies e Macromoléculas (SM Lab), Departamento de Física, Universidade Federal de Santa Maria, Santa Maria, RS 97105-900, Brazil

## Abstract

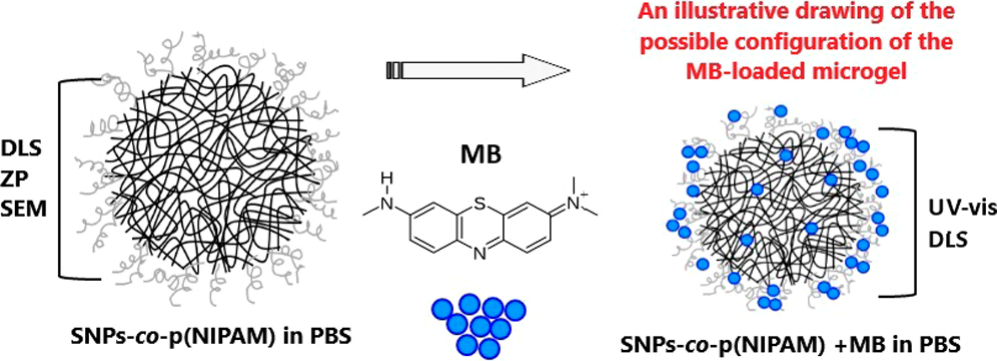

Hybrid microgels made from starch nanoparticles (SNPs) and poly(*N*-isopropylacrylamide) p(NIPAM) were used as promising hosts for the methylene blue (MB) dye. In this paper, these thermoresponsive microgels were characterized by dynamic light scattering (DLS), zeta potential measurements (ZP), and scanning electron microscopy (SEM) and evaluated as carriers for skin-targeted drug delivery. The hybrid microgel-MB systems in PBS solution were also studied by UV–vis spectroscopy and DLS, revealing discernible differences in spectral intensity and absorption shifts compared to microgels devoid of MB. This underscores the successful integration of methylene blue within the SNPs-*co*-p(NIPAM) microgels, signifying their potential as efficacious drug delivery vehicles.

## 1 Introduction

Approaches based on hydrogel particles derived from natural polymers with micro- and nanosized dimensions have been proposed as attractive drug delivery vehicles.^1,2^ Such structures are considered innovative drug release systems due to their excellent biocompatibility and extracellular matrix mimicry.^3^ Besides that, the hydrogels used in drug delivery systems can decrease the frequency of drug administration, hence improving patient compliance.^4^ The controlled drug release can generate a stable steady state drug concentration, avoiding the peak and valley phenomenon. This is beneficial to reduce toxicity and side effects. In addition, local release allows the maximum concentration of the drug molecules to be transmitted near the target, thereby reducing the dosage and drug toxicity.^5^

Hydrogels present a three-dimensional cross-linked structure composed of hydrophilic polymers able to take up large amounts of water without dissolving,^4,6,7^ which is possible due to the presence of hydrophilic groups, such as hydroxyl (−OH), amine (−NH_2_), carboxylic (−COOH), and sulfate (−SO_3_H) in the polymer structure.^4^ The water content fills up the mesh (or pores), allowing selective diffusion of solutes through the hydrogel polymeric matrix, which can be increasingly faster when the hydrogel has colloidal dimensions, as microgels.^6,8^

Smart microgels are chemically or physically cross-linked structures that can reversibly swell and deswell in response to various chemical and physical external stimuli such as temperature, pH, and light, making these structures a suitable reservoir in drug delivery systems.^4,9^ Among the external stimuli, the temperature is the most popular for environment-sensitive delivery systems.^4,10^ Thermoresponsive microgels possess a polymer network that can swell and deswell in aqueous solution below or above a specific temperature, known as lower critical solution temperature (LCST) or upper critical solution temperature (UCST), respectively.^11^

A particular type of thermoresponsive polymer, based on poly(*N*-isopropylacrylamide) p(NIPAM), has gained significant attention and has been widely studied. This polymer is composed of hydrophilic amide (−CONH−) groups and hydrophobic isopropyl (−CH(CH_3_)_2_) side chains. It is well-known that p(NIPAM) exhibits an LCST at 32 °C in water,^2,12^ slightly lower than the human body temperature of ∼37 °C.^7^ Consequently, p(NIPAM) microgels present a volume phase transition temperature (VPTT), assuming a hydrophilic behavior below the transition temperature and a collapsed gel due to the significant hydrophobic interactions above it. These changes in the temperature cause changes in the swelling of p(NIPAM) microgels, seriously influencing the diffusion of solutes from the interior to outside the aqueous medium.^6^ These characteristics make p(NIPAM) an excellent candidate for biomedical applications, such as drug delivery carriers, tissue engineering scaffolds, and wound treatment skin dressings.^7,13,14^ However, p(NIPAM) microgels possess shortcomings such as low biodegradability, relatively low drug loading capacity, and a burst release of drug molecules. Improving the performance of such microgels has been a significant challenge in the past decade. One strategy is to produce p(NIPAM)-based microgels of suitable species, such as inorganic nanoparticles, organic self-assemblies, and other polymeric components.^7^

To improve the biocompatibility of p(NIPAM) microgels, natural and degradable starch nanoparticles (SNPs) were already applied as copolymers in these systems.^12^ According to Zhang and Zhuo,^15^ the hydroxyl groups of starch increase the number of hydrogen bonds in the hydrogel, creating a more stable hydration structure around the hydrophobic groups. In another study, hybrid microgels made of starch nanoparticles (SNPs) and p(NIPAM), the authors observed a core–shell structure above the critical solution temperature, having p(NIPAM) in the core and the SNPs in the shell. It was suggested that within the core, drugs can be included and, as a function of the temperature, can be released. The quantitative and directional release of the drug can improve the curative efficacy and relieve their side effects.^2,16^

This work used hybrid microgels made of SNPs and p(NIPAM) to encapsulate methylene blue (MB) dye. Methylene blue (C_16_H_18_CIN_3_S) is a low molecular weight hydrophilic and cationic phenothiazinium compound (319.85 g mol^–1^).^17,18^ Hydrophilic/lipophilic balance and a net positive charge on MB allow it to penetrate biological membranes easily. The positive charge and low molecular weight also enhance interaction with bacteria and mammalian cells.^19^ MB also presents photophysical properties, which turn this dye into a photosensitizer (PS).^17^ The MB dye is a Food and Drug Administration (FDA)-approved drug because it inactivates viruses and bacteria (in vitro) and kills malignant cells (in vivo).^20^

It is worth mentioning that prolonged exposure to MB, even in the dark, can cause side effects, such as anemia, shocks, nausea, hypertension, serotonin syndrome, tissue necrosis, asthma, and jaundice, in mammals. Additionally, in biological media, MB is quickly reduced to the leuco-methylene blue form, which is not photosensitive.^17^ MB can be loaded in drug delivery systems to overcome these issues to increase drug bioavailability, reduce its cytotoxicity to the body, sustain a controlled release, and prevent leuco-methylene blue formation.^17^

In this study, hybrid microgels based on p(NIPAM) and SNPs (SNP-*co*-p(NIPAM)) were synthesized and then dispersed in a phosphate-buffered saline solution (PBS). Their physicochemical properties were investigated by dynamic light scattering (DLS), zeta potential (ZP), and scanning electron microscopy (SEM). Afterward, systems containing microgels and MB as model drugs were developed and characterized by UV–vis spectroscopy aiming at analyzing the load and release drug content in the systems according to the temperature, as well as DLS.

## 2 Materials and Methods

### 2.1 Materials

Regular corn starch was donated by Ingredion Brasil (https://www.ingredion.com/sa/pt-br.html). Dimethyl sulfoxide (DMSO) and ethanol were used without further purification. *N*-Isopropylacrylamide (NIPAM, recrystallized in hexane), *N*,*N*′-methylenebis(acrylamide) (BIS), sodium dodecyl sulfate (SDS), ammonium persulfate (APS), and methylene blue (MB) were purchased from Sigma-Aldrich and used as received. Solutions were prepared using water from a Millipore Waters Milli-Q purification system. All other chemicals (for PBS solution preparation) were of analytical grade and were used as received.

### 2.2 SNP-*co*-p(NIPAM) Microgel Synthesis

SNP-*co*-p(NIPAM) microgels were synthesized as reported by Leite et al.^2^ Starch 2% (w/v) in DMSO/H_2_O (9:1 v/v ratio) was prepared under a magnetic stirrer for 2 h at 40 °C. After cooling to room temperature, the solution was sonicated for 1 min. Then, 1 mL was dropped in 20 mL ethanol at 900 rpm, stirred for 1 h, and purified through centrifugation. Finally, SNPs were dried at 40 °C for 24 h. For microgels synthesis, 0.300 g SNPs and 0.300 g NIPAM (SNP:NIPAM 1:1 w/w ratio) were added in a three-necked flask with 50 mL degassed milli-Q water at 80 °C and 400 rpm. After thermal equilibrium, SDS (0.0011 mol L^–1^) and BIS (0.0041 mol L^–1^) were added, and N_2_ was bubbled through the solution for at least 30 min before polymerization. Then, APS (0.0027 mol L^–1^) was added, and the precipitation polymerization (PP) or surfactant-free precipitation polymerization (SFPP) reaction was kept at 80 °C for 4 h. The mixture was cooled to room temperature overnight with a magnetic stirrer. Microgels were purified through three centrifugations/redispersions in milli-Q water and then lyophilized. Samples were labeled as SNPs/NIPAM/SDS (synthesis in the presence of surfactant) and SNPs/NIPAM (surfactant-free synthesis).

### 2.3 Preparation of Inclusion Systems between Temperature-Sensitive Hybrid Microgels and Methylene Blue

MB-loaded microgels were prepared according to the method proposed by Cohen^21^ and Ribeiro et al.^22^ Hybrid microgels (1 mg mL^–1^) were dispersed in a PBS solution. Separately, MB (7.10^–3^ g L^–1^) was also dissolved in a PBS solution. After hybrid microgel dispersion, MB solution was added at a 3:2 (microgel dispersion:MB solution) v/v ratio. The solutions were stirred at 25 °C for 24 h and kept shelter from light during the experiment. All experiments were performed in triplicate. Samples were labeled SNPs/NIPAM/SDS/MB (microgel synthesized in the presence of surfactant and loaded MB) and SNPs/NIPAM/MB (surfactant-free microgel and loaded MB).

### 2.4 Characterization

#### 2.4.1 Dynamic Light Scattering (DLS)

DLS measurements were carried out at 20 and 35 °C using a Brookhaven (BI200 M goniometer with a BI9000AT digital correlator) with a He–Ne vertical polarized laser (λ = 632.8 nm) at a fixed scattering angle (θ = 90°), coupled with a thermal bath. The pinhole aperture was fixed at 200 μm. Data were processed using CONTIN^23^ (size distribution and correlation function) and the cumulant method (for polydispersity index, PDI).^24^ Each sample was measured in triplicate at a 0.05 mg mL^–1^ microgel suspension in water, and the reported values were given as the mean hydrodynamic diameter (*D*_h_ ± sd, nm).

To determine the VPTT of microgels, the average hydrodynamic diameter (*D*_h_) of microgels as a function of the temperature, ranging from 10 to 37 °C with increments of 2 °C between 10 and 30 °C and with increments of 1 °C above 30 °C were carried out using a Zetasizer Nano ZS (Malvern Instruments, USA) equipped with a 4 mW He–Ne laser. Measurements were performed at a wavelength of 632.8 nm, using the detection angle of 173°. The reported values are the mean of three independent measurements, and the results are given as the mean hydrodynamic diameter (*D*_h_ ± s.d., nm).

#### 2.4.2 Zeta Potential (ZP)

The ZP was measured by a Malvern Zetasizer (Malvern Instruments, USA) at 20 and 35 °C. ZP was calculated using the Smoluchowski equation from the electrophoresis mobility and electric field strength.^25^ The value was recorded as the average of five measurements and reported as the mean ± s.d. (mV). All experiments were performed in triplicate.

#### 2.4.3 Scanning Electron Microscopy (SEM)

Hybrid microgel samples were observed by using an SEM instrument (EVO MA10, Zeiss, Germany). Two microliters of diluted hybrid microgel samples were placed in a glass coverslip attached to stubs, dried at room temperature, and sputtered with an Au layer. Particle size analyses of SEM images were performed using the ImageJ software.^26^

#### 2.4.4 UV–Vis Spectroscopy Analysis, Efficiency, and Loading Capacity of Inclusion Systems

Spectra of MB calibration curve, pure hybrid microgels, and hybrid microgels-MB systems were carried out in a UV–vis spectrometer (Cary 50, Varian) with a Peltier cell for temperature controller, using a quartz cell with an optical length of 1 cm, covering the 600–700 nm wavelength range. The maximum peak with minimum interference was centered at 664 nm, which allows a calibration curve to be obtained using linear regression that indicates the MB concentrations in the respective solution. The molar absorption coefficient (ε) of MB was obtained from the absorption spectra at different MB concentrations (ranging between 3.13 × 10^–5^ and 2.19 × 10^–5^ mol L^–1^), using the Lambert–Beer Equation^17^ (eq 1).
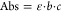
1where Abs is the absorbance, *b* is the optical path (1 cm), and *c* is the MB concentration.

From the stock solution of MB in PBS solution, aliquots were transferred to an optical cuvette, and the respective spectra were measured after each addition. Experiments were carried out in triplicate, and the measurements were performed at 20 and 35 °C.

Inclusion efficiency (% IE) and loading capacity (% LC) of MB in the microgels were also determined. The included MB and loading capacity percentage were determined from eqs 2 and 3.
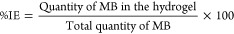
2
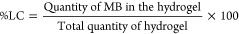
3

## 3 Results and Discussion

### 3.1 Characterization of Hybrid Microgels

**Figure 1:**
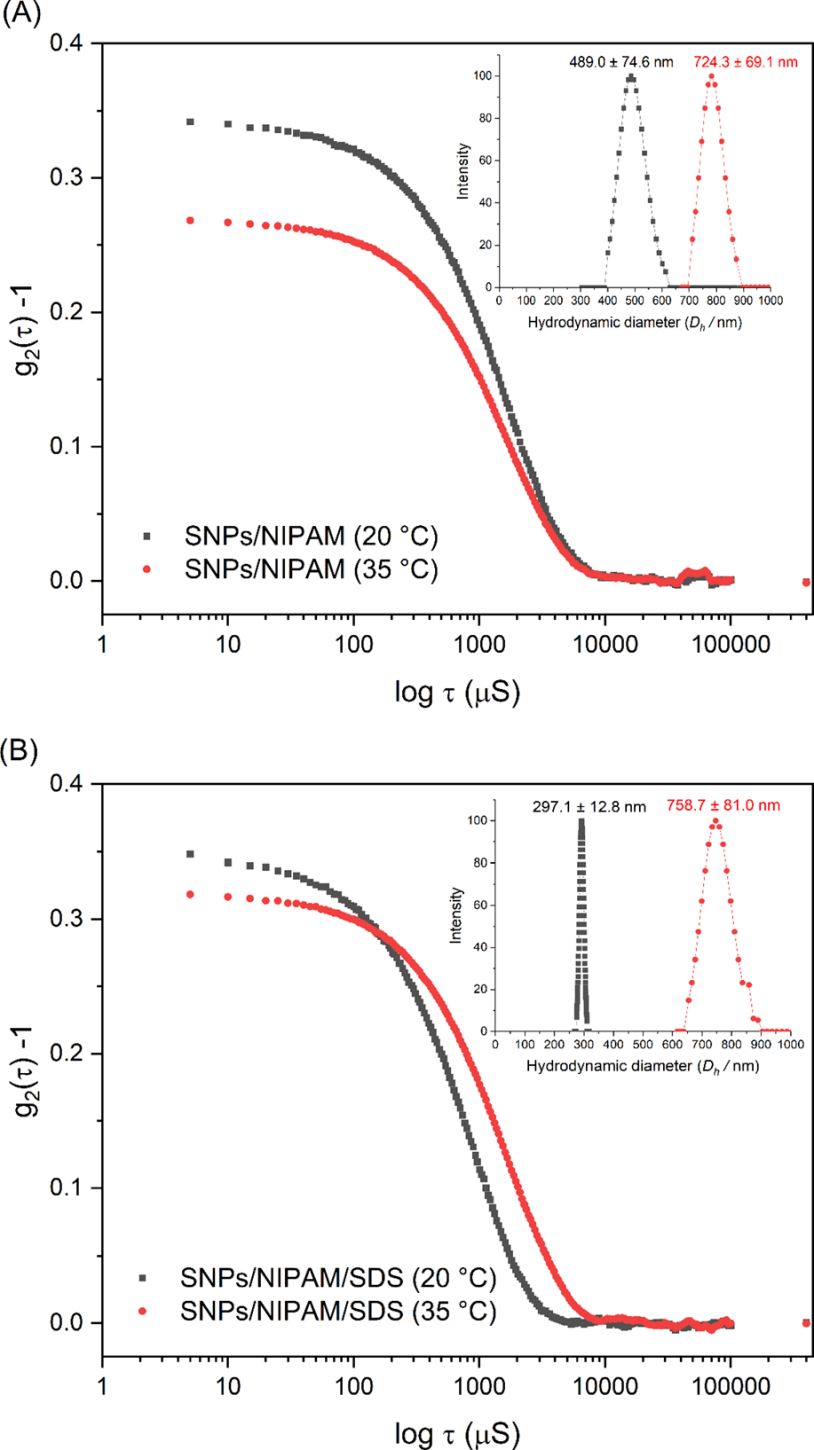
Correlation curves and CONTIN size distribution (*D*_h_, nm) (in detail, top right) for microgels (A) SNPs/NIPAM and (B) SNPs/NIPAM/SDS, at 20 °C (black square curve) and 35 °C (red circle curve). For the interpretation of the references to color in this figure legend, the reader is referred to the web version of this article.

Microgels were prepared via surfactant-free precipitation polymerization (SNPs/NIPAM) or precipitation polymerization (SNPs/NIPAM/SDS). Both microgels were analyzed for their particle size and distribution (Figure 1A, B).

The average particle size of microgels was measured at 20 °C (below VPTT) and 35 °C (above VPTT). These temperatures were also chosen to mimic the temperature of the drug delivery system before application (room temperature or close to it) and the temperature of drug release (skin temperature). Average values of *D*_h_ were obtained from CONTIN size distribution plots (Figure 1A, B—right top). The temperature increase above VPTT changed the position of these peak maxima. In both cases, *D*_h_ increased with increasing temperature, indicating the shrinking and, in this case, aggregation of the microgels.

According to the literature, the pNIPAM-based microgels are thermoresponsive systems. When the temperature is raised above the VPTT (above 32–33 °C for pure p(NIPAM) microgel in water), the polymer undergoes a phase transition.^2,12^ The swollen structure (hydrophilic state) collapses to a dehydrated structure (hydrophobic state).^27,28^ Although the NIPAM monomer is soluble in aqueous solutions at elevated temperatures, upon reaching a critical degree of polymerization, p(NIPAM) becomes increasingly hydrophobic, forcing an in situ rearrangement into higher-ordered morphologies composed of hydrophobic p(NIPAM) cores. This behavior is observed as a change from a transparent solution to an opaque dispersion, indicative of the coil-to-globule transition of p(NIPAM).^29^

Although the microgels were prepared in PBS, the results found in Figure 1 and in Table 1 below VPTT (20 °C) show the same trend found by Leite et al.,^2^ where the microgel synthesized with SDS showed smaller size and PDI when compared to the surfactant-free synthesis. Likely, SDS increases the number of formed nuclei due to a decrease in critical radius, leading to more but smaller particles. Thus, surfactants adsorb onto dispersed p(NIPAM) particles and SNPs, increasing their colloidal stability.^2,30^

**Table 1:** Average Intensity (kcps), Hydrodynamic Diameter (nm), Polydispersity Index (PDI), and Zeta Potential (mV) for SNPs/NIPAM/SDS and SNPs/NIPAM Microgels in PBS Solution, at 20 and 35 °C

	sample			PDI	
20	SNPs/NIPAM/SDS	76.9 ± 0.2	297.1 ± 12.1	0.020 ± 0.018	–5.33 ± 0.06
	SNPs/NIPAM	74.3 ± 3.3	489.0 ± 74.6	0.138 ± 0.030	–5.75 ± 0.63
35	SNPs/NIPAM/SDS	169.9 ± 6.3	758.7 ± 81.0	0.131 ± 0.030	–7.50 ± 0.03
	SNPs/NIPAM	170.5 ± 0.7	724.3 ± 69.1	0.140 ± 0.031	–9.74 ± 0.75

In the present study, the SNPs/NIPAM/SDS microgel dispersed in PBS solution also presented PDI values smaller than the SNPs/NIPAM microgel, as presented in Table 1. The PDI value is an important parameter, as it shows particle size uniformity. A small PDI value is desirable and indicates a narrow particle size distribution. It has been proven that a PDI value <0.2 is an indication of monodispersed particles.^31,32^

**Figure 2:**
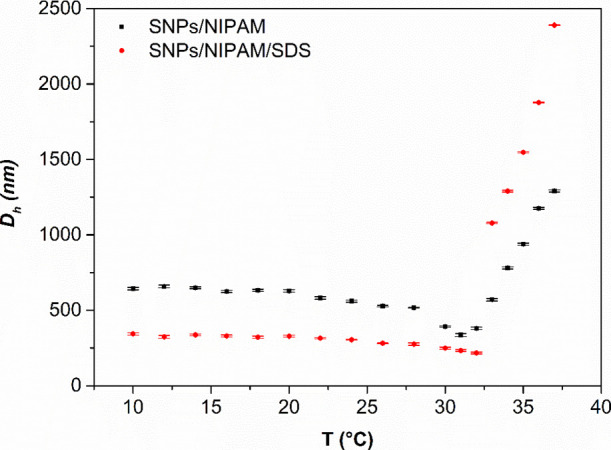
*D*_h_ versus temperature for microgels SNPs/NIPAM (black squares) and SNPs/NIPAM/SDS (red circles). For the interpretation of the references to color in this figure legend, the reader is referred to the web version of this article.

According to the literature, the transition temperature of the p(NIPAM) is slightly lower in PBS (around 32 °C, as can be seen in Figure 2) than in pure water, which means that in PBS solution, water–polymer interactions were replaced to some extent by the salt interactions with water and with the polymer, causing a “salting out” effect, well described by Hofmeister series^33^ for kosmotropic anions (HPO_4_^–^, H_2_PO_4_^2–^, and Cl^–^ present in PBS solution). The water has a greater tendency to solvate smaller particles. As a consequence of this effect, there was an increase in polymer–polymer interaction, reduced solubility in aqueous media, and an increase in the ionic strength of the solution.^33,34^

According to Figure 2, we observe the *D*_h_ quite the same in the temperatures ranging from 10 to 28 °C. From 30 to 32 °C, the diameter slightly decreases, followed by an increase in its apparent diameter according to the increase in temperature, showing the expected result for NIPAM-based microgels dispersed in PBS solution.

Besides comparable *D*_h_ values below VPTT for microgels dispersed in pure water^2^ and in PBS solution, the results presented in Figure 1 and Table 1 for measurements above VPTT (35 °C) are significantly shifted to higher size for both microgels. It is suggested that the salting out effect described previously caused by the PBS solution is responsible for this increase in *D*_h_. Otulakowski et al.^33^ carried out a study of thermoresponsive polymers in salt and PBS solution. For p(NIPAM) in PBS solution, at 0.5 g L^–1^, they found an increase in *D*_h_ above the transition temperature, wherever the heating step was abrupt or gradual. In an abrupt heating, there was a reduced contact time of the intrachain polymer–polymer interaction, and thus, the likelihood of aggregation decreased. In gradual heating, as presented in Figure 2, an increased contact time results in a stronger intrachain polymer–polymer interaction. As a result, the increased contact time promotes aggregation to larger sizes due to each heating equilibrium step.

Besides the differences between our work and the one reported by Otulakowski et al.,^33^ similar tendencies were found. For SNPs/NIPAM/SDS and SNPs/NIPAM microgels, abrupt heating to 35 °C increased the *D*_h_. It resulted in a larger size distribution curve, even though the PDI indicates a monodisperse size distribution in both cases. Evidence of microgel aggregation is observed in the red correlation curves of Figure 1, where some inhomogeneities were observed at the end of the relaxation time. According to Kang et al.,^34^ adding anions can also induce aggregate transition at a given temperature.

Another interesting fact about the DLS results is the scattering intensity, which is also reported in Table 1. Since the same concentration, aperture, and measurement parameters were fixed for both microgels, it is possible to compare the scattering intensity. The increase in temperature and the following phase transition due to the shrinking of microgel caused by polymer–polymer hydrophobic interactions causes an increase in the refractive index difference, resulting in higher scattering intensity. In this work, microgel shrinking was followed by particle aggregation.

Zeta potential results are also listed in Table 1. The zeta potential indicates the degree of repulsion between adjacent and similarly charged particles in dispersion.^35^ The zeta potential knowledge is valuable information on how hybrid microgel dispersed in PBS solution and MB may interact with each other.^36^ The particle surface charges are directly related to functional groups, and zeta potential values give the correlated estimation of the surface charges depending on the pH of the medium.^37^

According to ZP data presented in Table 1, under neutral conditions, both hybrid microgel particles present negative zeta potential values in the analyzed temperatures, which can be attributed to hydroxyl groups of SNPs at the particle’s surface. Besides that, the values are different from those obtained by Leite et al.^2^ According to the literature the ionic strength of PBS buffer is around 150 mM^38^ and the increase of the microgel hydrodynamic diameter can be explained by the formation of aggregates due to the ionic screening of the particle electrical charges. It was also observed that the increase in the temperature caused an increase, in modulus, in the electrostatic surface potential in both samples; i.e., at 35 °C the ZP values become more negative, indicating that the p(NIPAM) core collapses, although increasing *D*_h_ but presenting colloidal stability, where the surface is probably composed by SNPs with −OH groups. Both of the microgels at 35 °C had a lower zeta potential at pH 7.4 compared to the microgels at 20 °C, which could indicate their possible use in biomedical applications.^39,40^

**Figure 3:**
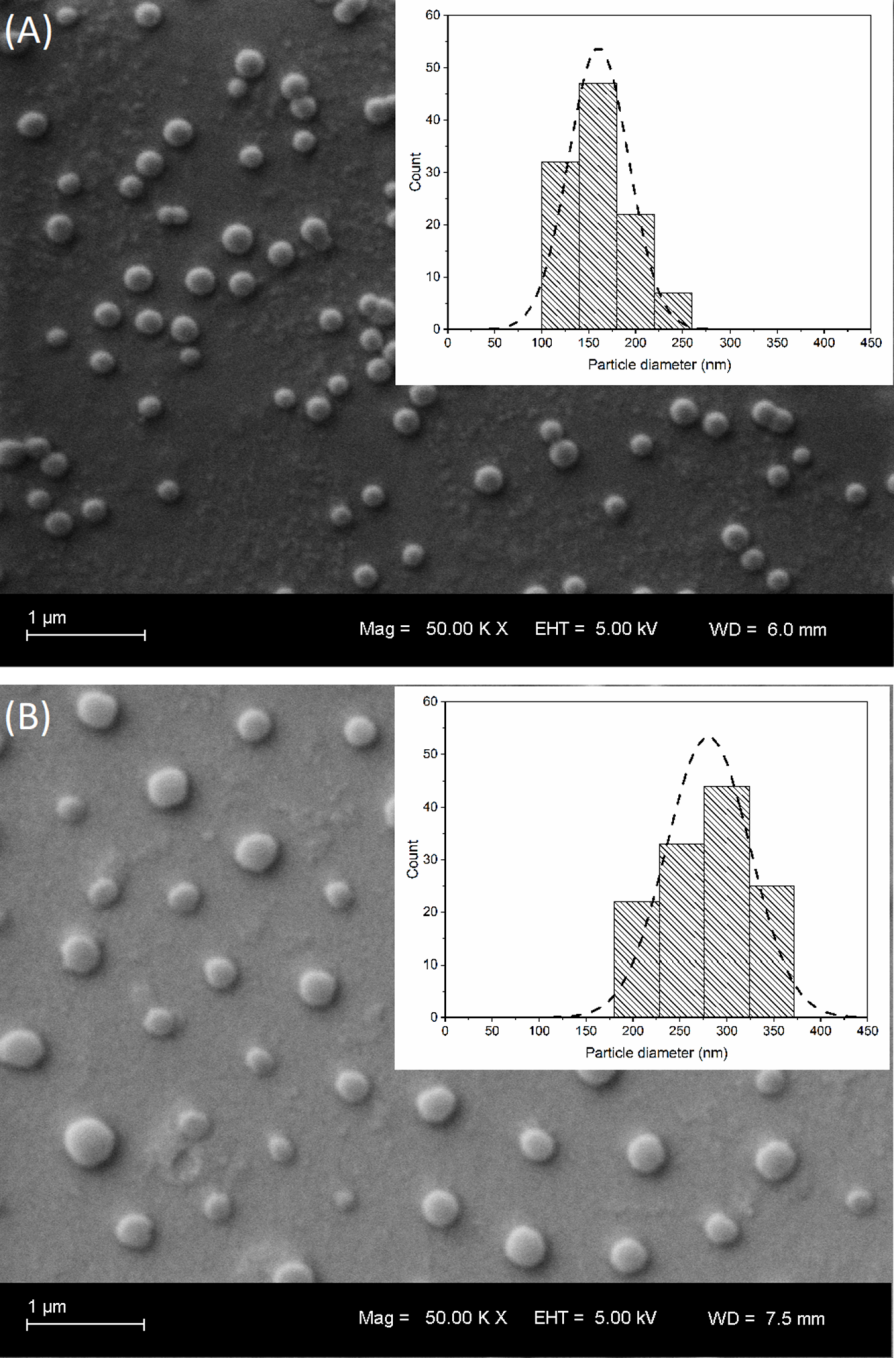
SEM of the hybrid microgels. In (A), SNPs/NIPAM/SDS (average *D*_h_ of 160 nm); in (B), SNPs/NIPAM (average *D*_h_ of 270 nm); in detail, particle size analysis using ImageJ.

SEM images (Figure 3) present the different hybrid microgels according to composition and confirm the results found in DLS analyses.

Both microgels (SNPs/NIPAM/SDS and SNPs/NIPAM) exhibit most likely spherical morphology, smoothed surfaces, and no aggregation, with average particle sizes of 160 and 270 nm, respectively. These are smaller than the diameters obtained by DLS in solution, which is expected since DLS measures the hydration radius, while the samples measured by SEM are in the dry state.^41^ Once again, as shown in the PDI results, a broad size distribution was observed for microgels synthesized in the absence of surfactant (Figure 3B).

### 3.2 Characterization of Hybrid Microgel-MB Systems

MB was selected as a drug model to evaluate the drug loading of the microgels. MB is a cationic molecule^42^ expected to interact electrostatically with the oppositely charged functional groups of the microgels. Besides that, MB contains polar groups, which behave as hydrophilic groups and are also involved in interacting with the microgel via hydrogen bonds or dipole–dipole intermolecular interactions. The network with a highly porous structure in the microgel provides a larger specific surface area to enable more active sites to interact with MB molecules. It promotes faster adsorption with high adsorption capacity.^42^

**Figure 4:**
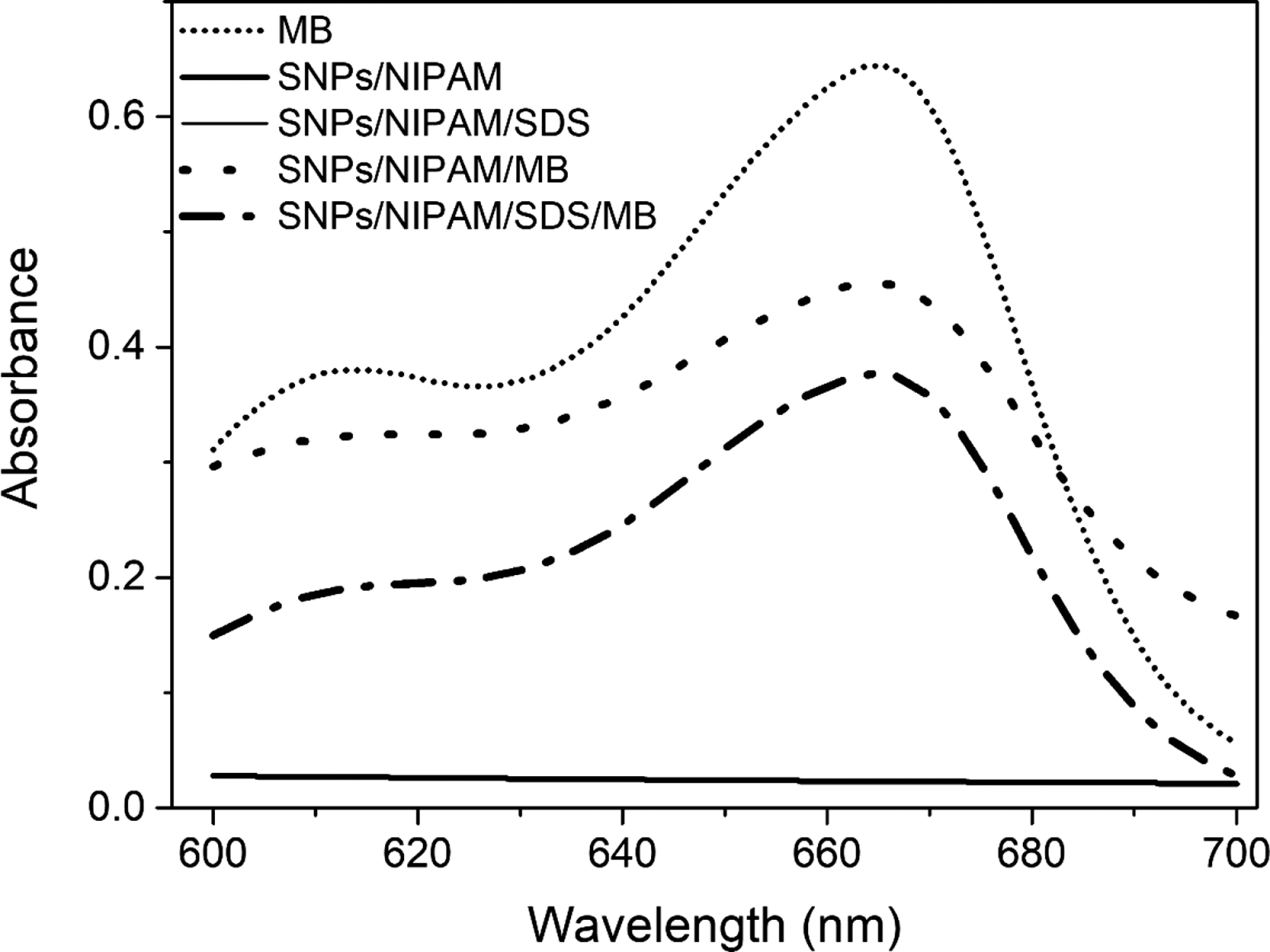
UV–Vis spectra, at 20 °C, of pure hybrid microgels (—), SNPs/NIPAM microgel loaded with MB (— · —), and SNPs/NIPAM/SDS microgel loaded with MB (−), and MB (···) in PBS solution at 20 °C.

The UV–vis spectrum of MB in a PBS solution has a strong absorbance at 664 nm (Figure 4). This band is assigned to the n → π transitions of monomeric MB photosensitizer^17^ and is the characteristic absorption of MB monomer (MB+). The shoulder peak at 615 nm is ascribed to the absorbance of the MB dimer.^43,44^ MB molar extinction coefficient in PBS solution was determined using the Lambert–Beer equation and presented the value of 2.9.10^–4^ L mol^–1^ cm^–1^, a similar value to that found by Silva and co-workers.^45^

Microgels possess polar functional groups that can adsorb and trap ionic dyes, such as MB. However, dye molecules may or may not penetrate microgels, depending on physical–chemical interactions between dye molecules and polymer networks of microgel. Therefore, as MB dye has great applicability in the medical field, understanding its encapsulation in new systems becomes very relevant.

In this study, we observed the formation of the delivery system between the microgel and MB by a slight shift of the absorption spectrum compared with the spectrum of the MB pure samples (Figure 4). First, we observed, after the loading, a decrease in the absorbance of MB solution in both microgels. The decrease of absorbance of the absorption peak relative to pure MB suggested that the MB molecules adsorbed onto and/or into the microgels.

The decrease in absorbance was slightly more significant for the microgel whose synthesis was carried out with surfactant. This can be explained by the presence of the SDS used during the synthesis. The SDS adsorbs onto and/or into dispersed pNIPAM particles, increasing their colloidal stability.^2^ Thus, the microgel structure becomes more organized, allowing MB to be adsorbed more efficiently. In the work by Leite et al.,^2^ microgels synthesized with SDS showed a higher organization order, studied by small-angle X-ray scattering (SAXS).

Besides that, the ratio of dimer absorbance to monomer absorbance in the hybrid microgel-MB systems was slightly larger than that of the MB solution, indicating the occurrence of dimerization of some MB molecules. On the other hand, the maximum absorption peak observed was unchanged. This result indicated that the MB monomer was the predominant form in the complex, and some dimer forms were also present. This fact was already observed in other studies where MB was associated with other molecules.^46−49^

MB is a positively charged molecule that can interact strongly with negatively charged moieties,^50^ i.e., with the microgels’ functional groups (mainly −OH, −NH, −CO). In this study, the two synthesized hybrid microgels present −OH groups of the SNPs. It is suggested that the hydroxyl group can promote electrostatic attractive forces with positively charged MB. As a result, MB molecules can also be adsorbed on the SNP chains. In addition, the pNIPAM primary interaction at 20 °C is characterized by the amide moieties on its side chain in the polymer structure.^51^ Then, the adsorption driving force is also related to the electrostatic interaction between the amide groups of the microgels and positively charged MB.

Inclusion efficiency and loading capacity of the drug are important aspects for microgels applied to delivery systems. Surfactant-free microgels, at 20 °C, presented lower values of inclusion efficiency and loading capacity than microgels synthesized in the presence of surfactant (Table 2).

**Table 2:** Inclusion Efficiency (%) and Loading Capacity (%) of Hybrid Microgels-MB Systems at 20 and 35 °C

***T*** (°C)	**sample**	**inclusion efficiency** (%)	**loading capacity** (%)
20	SNPs/NIPAM/SDS/MB	32.8 ± 0.3	71.9 ± 0.7
	SNPs/NIPAM/MB	16.4 ± 0.5	35.9 ± 1.2
35	SNPs/NIPAM/SDS/MB	20.3 ± 1.3	18.7 ± 0.9
	SNPs/NIPAM/MB	20.4 ± 0.5	44.6 ± 4.3

Entrapment efficiency and drug loading capacity are essential for microgels used in delivery systems in order to optimize drug dosage. The microgel synthesized via the SFPP route presented lower entrapment efficiency and loading capacity values in comparison to microgels synthesized via the PP route. Moreover, the presence of a surfactant led to an increase in the loading capacity of the system. It can be asserted that employing microgels prepared via SFPP to encapsulate MB is unfavorable. As Table 2 demonstrates, with increasing temperature, the inclusion efficiency and loading capacity values decrease only for the microgels prepared via PP. As the kinetic energy of the microgels increases with the increasing temperature, the MB molecules may not leave the microgel structure in the SNPs/NIPAM/MB system. On the other hand, as the structure is more organized in the SNPs/NIPAM/SDS microgel, the MB will be more easily released from the microgel structure.

**Figure 5:**
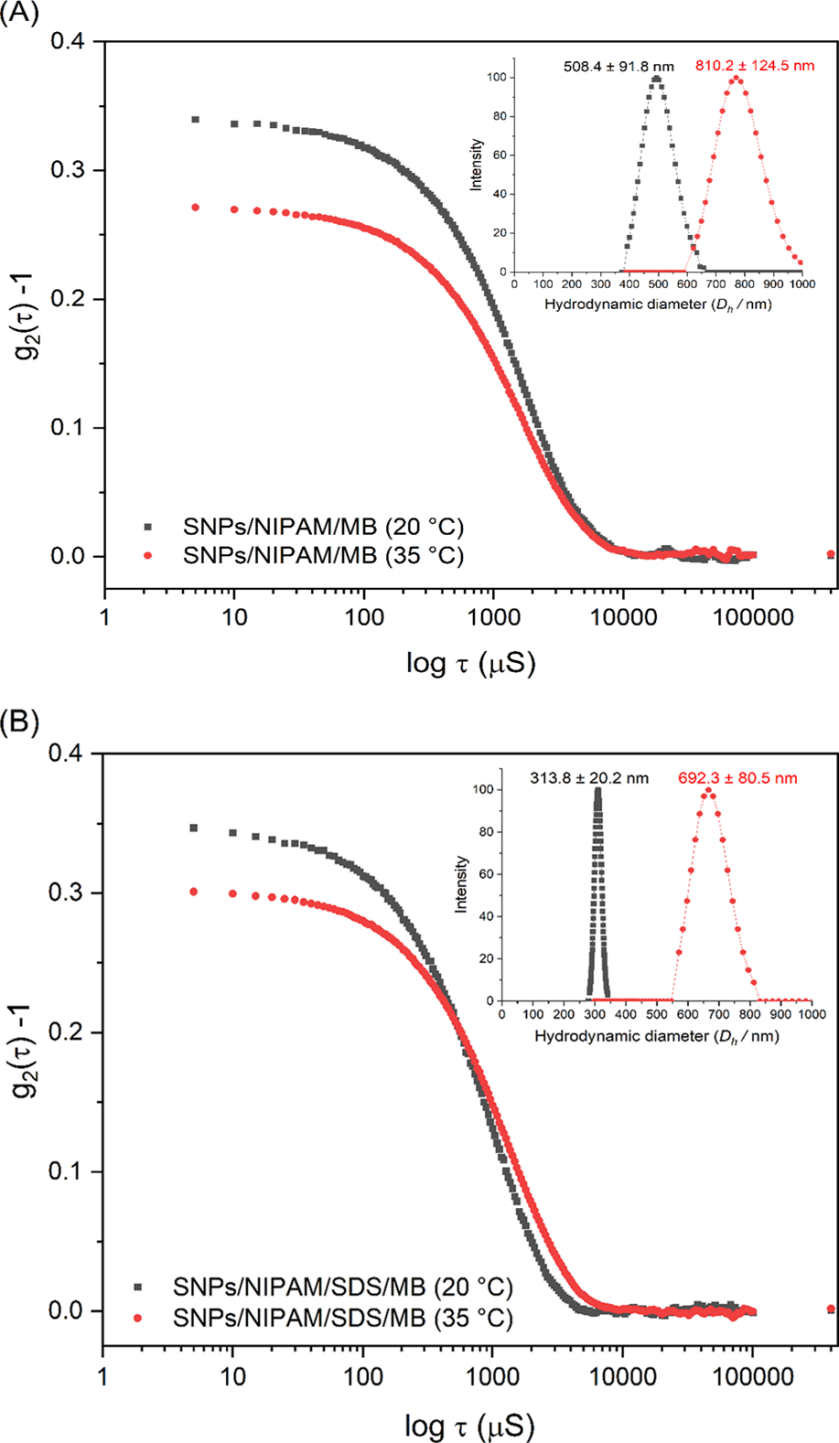
Correlation curves and size distribution (*D*_h_, nm) (in detail, right top) for microgels (a) SNPs/NIPAM/MB and (b) SNPs/NIPAM/SDS/MB at 20 °C (black square curve) and 35 °C (red circle curve). For the interpretation of the references to color in this figure legend, the reader is referred to the web version of this article.

**Figure 6:**
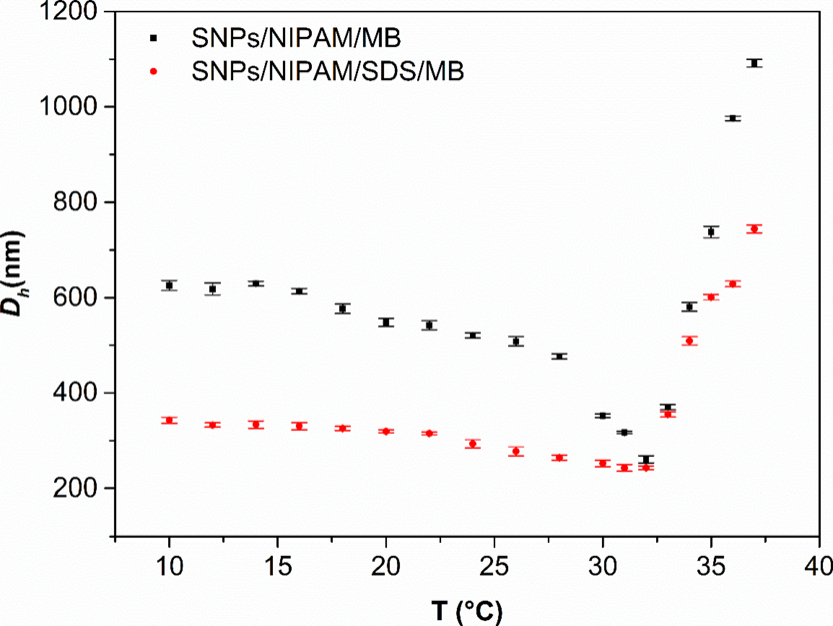
*D*_h_ versus temperature for microgels SNPs/NIPAM/MB (black squares) and SNPs/NIPAM/SDS/MB (red circles). For interpretation of the references to color in this figure legend, the reader is referred to the web version of this article.

The particle size distribution of the microgel with MB dye is shown in Figure 5 and Table 3, and the *D*_h_ versus temperature is shown in Figure 6. The adsorption of MB molecules onto microgels did not affect the overall DLS results, and similar intensities, *D*_h_, PDI, and behavior according to the increasing temperature were found. As can be seen, the *D*_h_ of microgels loaded with MB was slightly larger than that of microgels without the dye (Table 1). However, only the SNPs/NIPAM/SDS/MB microgel has its *D*_h_ statistically different (at a 95% confidence interval level using a two-sample *t* test) from the *D*_h_ of the SNPs/NIPAM/SDS microgel, measured at 20 °C. Such a difference can be related to the narrow size distribution of this microgel at this temperature. Also, it may be associated with a large amount of MB loaded at this temperature compared to the SNPs/NIPAM/MB microgel. Besides that, for both systems, it was observed that the sizes of microgels increased with the increase of the temperature, followed by an increase in the intensity of scattered light, just like observed for pure hybrid microgels.

**Table 3:** Average Intensity (kcps), Hydrodynamic Diameter (nm), and Polydispersity Index (PDI) for SNPs/NIPAM/SDS and SNPs/NIPAM Microgels in PBS Solution, at 20 and 35 °C

***T*** (°C)	**sample**	**intensity** (kcps)	*D***_h_** (nm)	**PDI**
**20**	SNPs/NIPAM/SDS/MB	72.8 ± 0.3	313.8 ± 20.2	0.043 ± 0.022
	SNPs/NIPAM/MB	67.6 ± 4.4	508.4 ± 91.8	0.219 ± 0.020
**35**	SNPs/NIPAM/SDS/MB	172.5 ± 4.7	692.3 ± 80.5	0.162 ± 0.019
	SNPs/NIPAM/MB	158.9 ± 5.2	810.2 ± 124.5	0.170 ± 0.022

**Figure 7:**
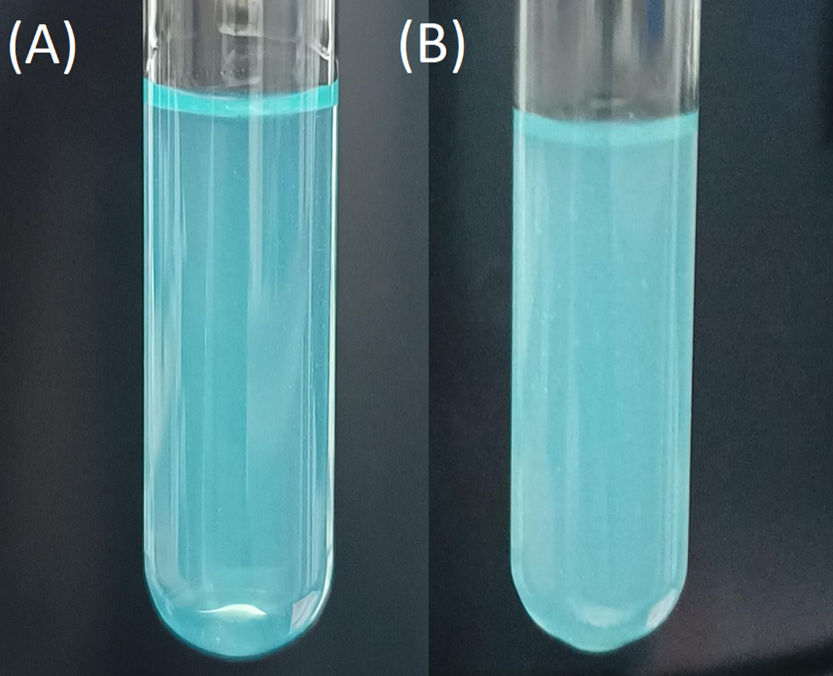
Images of the SNPs/NIPAM/SDS/MB microgel at 20 °C (A) and at 35 °C (B), at the preparation concentration.

Furthermore, the increased temperature also caused an increase in the refraction index difference, such as the microgel without MB (Figure 7). Figure 7 presents the SNPs/NIPAM/SDS/MB system at 20 °C (Figure 7A) and at 35 °C (Figure 7 B), right after the 24 h of preparation. With increasing temperature above VPTT, the breaking of hydrogen bonds between water and polar amide groups of p(NIPAM) occurs under the Brownian motion influence and intensification of hydrophobic interactions of p(NIPAM) isopropyl groups, easily seen by the turbidity of the system.^52^

Finally, all the samples exhibited particle sizes at 20 °C and narrow distribution (even at 35 °C) that are adequate for penetration across cellular barriers and retention at the site of action, crucial factors for drug delivery.^53^

## 4 Conclusions

Hybrid microgels made from SNPs and p(NIPAM) were used as hosts of the MB dye. These microgels are temperature-sensitive and were evaluated as carriers for skin-targeted drug delivery. DLS studies in PBS solution (used to imitate physiological pH) have shown that the microgels synthesized with SDS showed a smaller size and PDI when compared to the free-surfactant synthesis, as expected. Besides that, an increase in the temperature caused an increase in the size of the microgels. It led to subsequent particle aggregation, a completely different behavior from microgels dispersed in water above VPTT.

The results of the UV–vis analysis demonstrated that it was possible to incorporate and release MB in the SNPs-*co*-pNIPAM microgels (especially for the microgel prepared via the precipitation polymerization route), and the microgels present an adequate size for in vivo applications. In brief, the hybrid microgels proved promising candidates for encapsulating the MB dye. However, more studies concerning the structure, and location of MB in microgels as well as release and kinetic studies, and incorporation of MB used for different therapeutic applications is necessary.
